# Decoupling Mechanical and Conductive Properties of Cellulose Ionogels for Flexible Electronics: A Review

**DOI:** 10.3390/gels12050440

**Published:** 2026-05-17

**Authors:** Zhixuan Yang, Shuailin Li, Youjia Yang, Jiawei Yang, Ruiying Zhang, Jianguo Li, Bin Chen

**Affiliations:** School of Materials Engineering, Fujian Agriculture and Forestry University, Fuzhou 350108, China

**Keywords:** cellulose ionogels, flexible electronics, mechanical robustness, ionic conductivity, performance decoupling

## Abstract

High-performance flexible electronics require soft materials that combine mechanical robustness with efficient ionic conduction. In conventional ionogels, however, these requirements often conflict: dense networks improve strength but reduce the free volume and mobility needed for ion transport. This review provides a critical overview of recent progress in cellulose-based ionogels, with emphasis on design principles for decoupling mechanical and conductive properties. We discuss how cellulose precursors, crosslinking architectures (hydrogen bonding, covalent networks, and metal-ion coordination), and processing histories determine gel structure and mechanical integrity. We then highlight strategies that mitigate the trade-off, including precursor engineering, phase-separated networks, double-network architectures, crystallization-induced reorganization, and anisotropic assembly. Representative applications in flexible sensors, flexible energy-storage devices, and soft actuators are also summarized. This review offers a practical framework for designing cellulose-based soft functional materials with robust mechanics and sustained ionic conductivity.

## 1. Introduction

**Figure 1:**
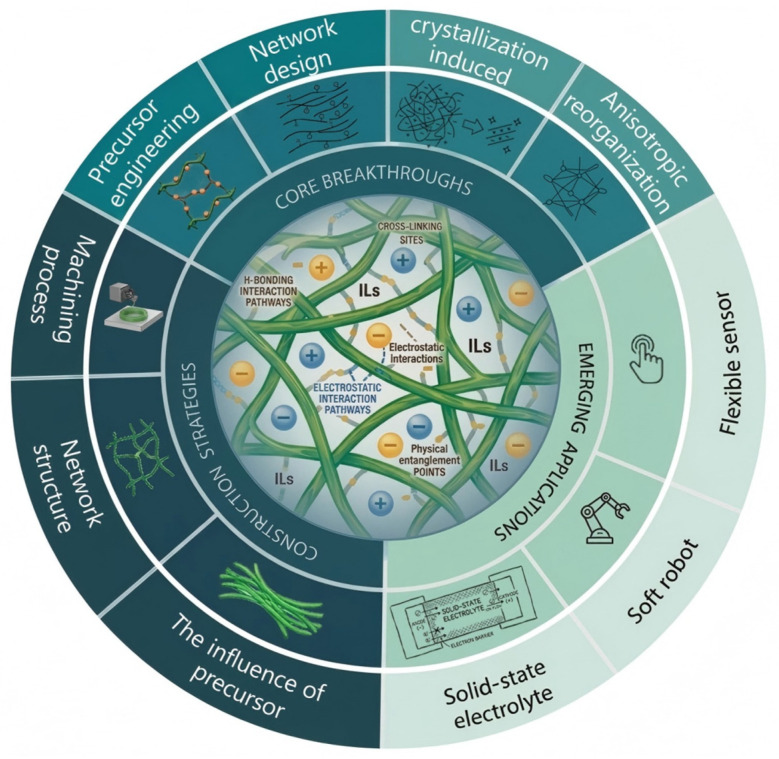
Schematic illustration of the design logic, key breakthroughs, and emerging applications of high-strength cellulose ionogels.

Flexible electronics play an important role in health monitoring, smart healthcare, and human–machine interaction. As the field advances rapidly, the performance requirements for flexible materials have become increasingly demanding. To support high-performance devices, these materials must maintain stable charge transport under large deformation and repeated mechanical loading, thereby ensuring reliable operation [1,2,3,4,5,6,7,8,9]. In addition to intrinsic flexibility, they therefore need sufficient mechanical robustness and dependable electrical performance. For conventional soft materials, however, these requirements are often difficult to satisfy simultaneously because strategies that increase mechanical strength frequently compromise conductivity. As a result, developing soft functional materials that can partially decouple mechanical reinforcement from electrical performance has become a major focus in flexible electronics (Figure 1).

**Figure 2:**
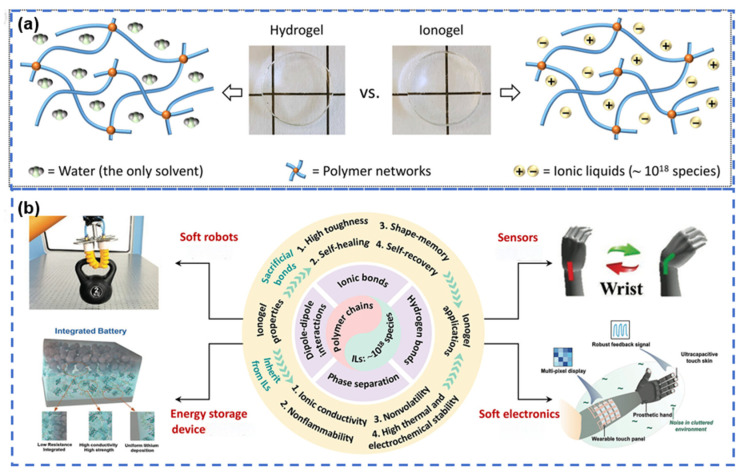
Overview of ionogels for flexible electronics and the design significance of cellulose-based ionogels. (**a**) Schematic illustration of the structural difference between hydrogels and ionogels [15]. (**b**) Key physicochemical features, interaction mechanisms, and typical applications of ionogels [15].

Hydrogels have attracted broad interest in flexible electronics because their water-swollen polymer networks provide mechanical compliance, ionic conductivity, and good biocompatibility [10]. Their long-term stability, however, is limited by water. Evaporation can cause performance drift, whereas dehydration and freezing reduce reliability in complex environments [11]. Aqueous systems also generally have a narrow electrochemical stability window [12,13]. Ionogels address these limitations by confining ionic liquids within solid or semisolid polymer networks. They retain the low volatility, high thermal stability, and wide electrochemical window of ionic liquids, while showing better environmental stability than conventional hydrogels (Figure 2) [14,15]. Nevertheless, ionogels still face an inherent trade-off between mechanical strength and ionic conductivity. Mechanical reinforcement typically requires higher crosslinking density, stronger intermolecular interactions, and lower free volume, whereas efficient ion transport depends on segmental mobility, well-developed ionic domains, and continuous conductive pathways [16,17,18,19].

Among the matrix materials used to construct ionogels, cellulose is especially attractive because it is abundant, renewable, biocompatible, and rich in hydroxyl groups for chemical modification [20]. Cellulose also offers hierarchical tunability across molecular chains, microfibrils, and network assemblies, giving it broad design flexibility over multiple length scales [21]. These features make cellulose a promising scaffold for ionogels [22]. Its multilevel structure provides opportunities to decouple mechanical strength from ionic conductivity, making cellulose one of the most useful platforms for balancing these competing properties in ionogel systems [23].

Although cellulose-based materials and ionogels have been widely reviewed, the design principles that govern the trade-off between mechanical robustness and ionic conductivity in cellulose ionogels remain insufficiently summarized. Most existing reviews emphasize gel chemistry, material categories, or specific applications, whereas the physicochemical basis for decoupling mechanical performance from ion transport has received less attention. Here, performance decoupling refers to structural design that mitigates the conventional trade-off by spatially, dynamically, or temporally separating load-bearing and ion-transport functions. From this perspective, this review focuses on how rational structural design can balance mechanical robustness with sustained ion transport. We first discuss how cellulose characteristics, network architecture, and processing strategies establish the structural basis of mechanically robust ionogels. We then summarize mechanisms that alleviate the mechanical-conductive trade-off by coordinated regulation of the load-bearing network and ionic-transport environment. Finally, we highlight representative applications, discuss remaining challenges, and outline future directions for cellulose ionogels in next-generation flexible electronics.

## 2. Design Principles of Mechanically Robust Cellulose Ionogels

The mechanical performance of cellulose ionogels is governed mainly by three interrelated factors: the cellulose source, the mode of network construction, and the processing conditions. Together, these factors shape the structural integrity of the gel matrix and its capacity to confine ionic liquids. This section, therefore, outlines the main structural design principles for constructing cellulose ionogels with high mechanical robustness.

### 2.1. Effect of Cellulose Precursors on the Structural Formation of Ionogels

**Figure 3:**
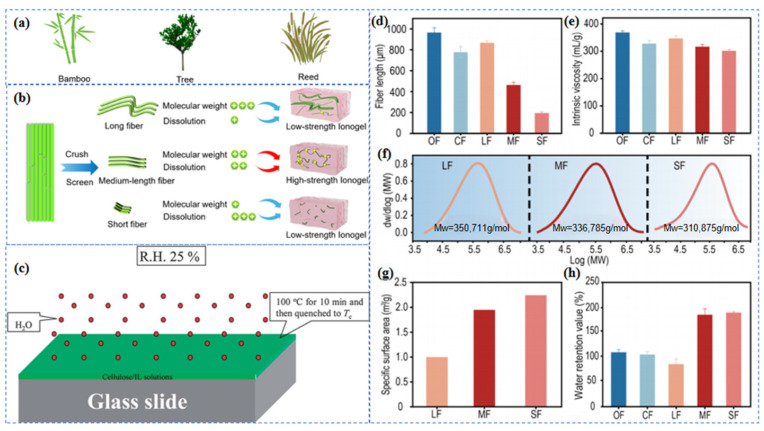
Schematic illustration of the effects of cellulose precursor characteristics on the structural formation of cellulose ionogels. (**a**) Photographs of raw materials (bamboo, tree, reed) [22]. (**b**) Schematic illustration of ionogel preparation from fibers of different lengths (long/medium/short) [22]. (**c**) Image showing preparation and morphology of different types of cellulose spherulites from concentrated cellulose ionic liquid solutions [27]. (**d**,**e**) Bar charts comparing the fiber length and index viscosity of regenerated celluloses [22]. (**f**) Molecular weight distribution of medium-length fiber (MF) derived cellulose [22]. (**g**,**h**) Bar charts showing the specific surface area and water retention value of celluloses from different sources [22].

The intrinsic characteristics of cellulose precursors fundamentally determine their dissolution kinetics, subsequent network assembly, and the final gel structure [24]. More specifically, factors such as cellulose source, molecular weight, and initial morphology influence polymer chain mobility, intermolecular interactions, and the continuity of the regenerated matrix [25]. Therefore, the rational selection of precursors is crucial for balancing processability, structural integrity, and ionic transport efficiency, thereby optimizing overall performance (Figure 3a,b,d–h) [22,26,27].

#### 2.1.1. Cellulose Precursors

Cellulose precursors obtained from wood pulp, cotton fibers, agricultural residues, and bacterial sources differ markedly in purity, crystalline organization, and fibrillar structure. These differences strongly affect the structure-property relationships of the resulting ionogels. They influence not only cellulose dissolution and regeneration but also the ability to regulate mechanical reinforcement and ion-transport pathways independently [28,29]. Plant-derived pulp is abundant and readily processed, but its structural heterogeneity can limit precise control over conductive channels. Cotton-derived cellulose has higher purity and a more ordered supramolecular structure, making it well suited for mechanically robust networks; its ionic conduction can also be tuned during regeneration [30]. Bacterial cellulose is particularly valuable because it already contains a continuous nanofibrillar network that can be directly used in ionogel fabrication. In such systems, the preserved interconnected framework supports mechanical integrity while maintaining continuous ion-transport pathways. The choice of cellulose precursor is therefore a key design variable for decoupling mechanical strength from ionic conductivity and optimizing overall ionogel performance [31,32].

#### 2.1.2. Cellulose Molecular Weight

Cellulose molecular weight is closely related to precursor type and processing history, and it strongly affects both cellulose dissolution and network reconstruction after dissolution [33]. Higher-molecular-weight cellulose generally has longer chains and a greater tendency to entangle, which favors the formation of regenerated networks with better mechanical properties and greater structural continuity. Excessively high molecular weight, however, can reduce dissolution efficiency and markedly increase viscosity, leading to incomplete dissolution, structural heterogeneity, and even premature gelation [29,34]. Low-molecular-weight cellulose is generally easier to process, but the resulting networks often show weaker cohesion and lower load-bearing capacity. Recent studies indicate that the relationship between molecular weight and material performance is not linear; instead, it is constrained at both the high- and low-molecular-weight extremes. At the high end, increasing molecular weight initially promotes chain entanglement, but further chain orientation becomes hindered once a critical range is exceeded [35]. At the low end, reducing the degree of polymerization can substantially alter the regenerated structure and ultimately affect the overall performance of flexible materials [36]. Taken together, these findings suggest that high-strength cellulose ionogels do not result from simply maximizing molecular weight. Rather, an appropriate molecular-weight range must be identified to balance processability with structural requirements. Such a range should allow controllable dissolution and the formation of a continuous, robust network while avoiding an excessive reduction in the free volume required to accommodate ionic liquids.

#### 2.1.3. Cellulose Precursor Morphology

Cellulose precursor morphology is another key factor controlling ionogel mechanics. Cellulose can be fully dissolved and regenerated to form a gel network, or it can be incorporated as structural units with defined morphologies, such as nanocrystals, nanofibrils, short fibers, or bacterial cellulose. Fully dissolved cellulose reinforces the matrix mainly through chain entanglement and the reformation of intermolecular interactions. By contrast, rigid nanocrystals usually act as discrete reinforcing units, whereas nanofibrils, short fibers, and intact bacterial cellulose are better at forming continuous support frameworks and stress-transfer pathways [37]. In one representative study, nanocellulose served as a macromolecular covalent crosslinker for ionogel construction, producing a tensile strength of 3.29 MPa, a toughness of 13.15 MJ m^−3^, and an elongation at break of 975% [38]. In concentrated MCC/AMIMCl (microcrystalline cellulose/1-allyl-3-methylimidazolium chloride) systems, microcrystalline cellulose can form spherulites and radially oriented fibrillar textures during regeneration, with size and morphology controlled by concentration and crystallization conditions (Figure 3c) [27]. Overall, precursor morphology strongly affects the spatial distribution and connectivity of ion-transport pathways. Discrete nanocrystals can enhance interfacial transport, whereas continuous fibrous or networked morphologies are more suitable for long-range conductive pathways. This morphology-dependent regulation provides a structural basis for combining relatively high stiffness with efficient ion transport.

### 2.2. Effect of Cellulose Network on Ionogel Structure

Once the cellulose precursor has been selected, constructing a continuous cellulose-based network scaffold becomes a central factor in ionogel performance. The final network architecture determines the matrix’s ability to dissipate stress, maintain structural integrity, and preserve the spatial continuity of ionic-liquid-rich domains. Because a single network often cannot simultaneously provide sufficient strength, toughness, and structural stability, architectures that incorporate multiple interactions or hierarchical features have become an important strategy for developing mechanically robust cellulose ionogels.

#### 2.2.1. Hydrogen-Bonded Network

Hydrogen-bonded physical networks form the most basic structural framework in cellulose ionogels because cellulose backbones contain a high density of hydroxyl groups. During dissolution, regeneration, and solvent exchange, these groups promote intermolecular interactions and help establish the initial network structure [39]. Because these hydrogen bonds are reversible, they can act as sacrificial junctions that dissipate mechanical energy during deformation and enable partial structural recovery, which is advantageous in applications involving repeated stretching, self-recovery, and strain sensing [40]. This reversibility has enabled the development of cellulose ionogels with both rubber-like stretchability and excellent low-temperature tolerance through the combined effects of intrinsic hydrogen bonding and ionic-liquid-mediated chain organization [41].

Recent studies quantitatively confirm the role of hydrogen-bonded networks in regulating the mechanical-conductive balance of cellulose ionogels. In a cellulose/[Bmim]Cl (1-butyl-3-methylimidazolium chloride)/H_2_O dynamic gel, water-regulated hydrogen-bond topology increased tensile strength and toughness from 0.30 MPa and 246.2 J·m^−2^ to 3.50 MPa and 1652.9 J·m^−2^, while ionic conductivity reached 40 mS cm^−1^ at 32 wt% H_2_O [39]. Reconstructed cellulose ionogels showed enhanced cellulose-cellulose hydrogen bonding and weakened cellulose-ion electrostatic adsorption, giving a tensile strength of 3.5 MPa, an ionic conductivity of 14.3 mS·cm^−1^, and a voltage window of 3.0 V [40]. Molecular-configuration engineering further enabled cyanoethylated cellulose ionogels to reach > 990% strain, 1.8 MPa tensile strength, 21.35 mS cm^−1^ ionic conductivity, and a Seebeck coefficient of ~68 mV·K^−1^ through reconstructed hydrogen bonds between residual -OH and -CH_2_CH_2_CN groups [41]. In PAM (polyacrylamide)/cellulose semi-IPN (semi-interpenetrating polymer network) ionogels, FTIR (Fourier Transform Infrared Spectroscopy) confirmed hydrogen bonding between PAM carbonyl groups and cellulose hydroxyl groups, while LF-NMR (low-field nuclear magnetic resonance) showed that cellulose increased crosslinking density from 7.433 × 10^−6^ to 1.41 × 10^−4^ mol·cm^−3^. This restricted segmental mobility, reduced swelling, and produced 3109% strain, 4861 kJ m^−3^ toughness, and 1.76 mS·cm^−1^ conductivity [42]. These results show that reversible hydrogen bonds can dissipate energy, while suitable crosslink density and weakened ion-binding interactions help maintain continuous ion-transport pathways.

Despite these advantages, hydrogen-bonded physical networks still tend to suffer from creep, limited dimensional stability, and incomplete mechanical recovery at high ionic-liquid contents. These limitations highlight the need for complementary reinforcement strategies.

#### 2.2.2. Covalently Cross-Linked Network

Compared with purely hydrogen-bonded networks, covalently crosslinked networks provide a more stable framework, especially under sustained loading, high ionic-liquid contents, or large temperature fluctuations. Permanent covalent junctions anchor the matrix, suppress creep, preserve dimensional stability, and maintain network continuity when reversible intermolecular interactions are insufficient [43]. A representative example is the irradiation-induced crosslinking strategy reported by Kimura and co-workers, in which cellulose was converted directly into a gel in room-temperature ionic liquids under gamma-ray irradiation [44]. Ionizing radiation generated reactive radicals on cellulose chains, which then formed permanent intermolecular covalent linkages and converted the cellulose/ionic-liquid mixture into a chemically stabilized ionogel network. Later mechanistic studies showed that this process is closely related to hydroxyl-radical generation and can be promoted by water in the ionic-liquid medium [45]. These findings indicate that covalent network formation depends strongly on the reaction environment. Unlike reversible hydrogen-bonded networks, covalent crosslinking gives cellulose ionogels a more stable three-dimensional framework and greater resistance to structural relaxation or solvent-induced rearrangement, both of which are important for long-term ionic conduction.

Excessively high crosslinking density or fully static network structures, however, can reduce deformability and restrict the local segmental motion needed for ion transport. Dynamic covalent chemistry offers a way to address this limitation by preserving network integrity while allowing local bond exchange, structural rearrangement, and intrinsic damage repair [46]. Dynamic covalent linkages such as imine, disulfide, and boronate ester bonds can provide both durability and structural adaptability [47]. Although these motifs are not yet widely used in cellulose ionogels, related ionic-liquid gels based on boronate ester bonds have shown self-healing, recyclability, and plasticity [48]. Introducing dynamic covalent design into cellulose ionogels may therefore help overcome the brittleness and limited segmental mobility of static networks and provide a more adaptable route for advanced ionogels (Table 1).

#### 2.2.3. Metal-Ion-Coordinated Network

Coordination interactions and ionic crosslinking provide another important reinforcement mechanism in cellulose ionogels. In this approach, multivalent ions interact with intrinsic hydroxyl groups on cellulose or with grafted functional groups, forming crosslinking sites that are stronger and more directional than ordinary physical interactions while still retaining a degree of reversibility. This dynamic character makes ionically crosslinked gels attractive for balancing mechanical robustness with ionic conductivity because the framework can be reinforced without completely suppressing stress relaxation or local structural rearrangement [49]. The effects of coordination interactions depend strongly on ion species, binding strength, and the local network environment, so their role cannot be reduced simply to a higher crosslinking density [50].

Existing studies support the effectiveness of this tunable reinforcement strategy. For example, one study reported a Ca^2+^-coordinated cellulose ionogel with a tensile strength of 6.84 MPa and an ionic conductivity of 31.7 mS·cm^−1^ [23]. The material also retained excellent flexibility at temperatures as low as −196 °C, which was attributed to stress-induced molecular-chain orientation. In this system, ionic coordination not only reinforced the network but also helped tune the local microenvironment in ways that favored charge transport.

In summary, at the molecular-to-mesoscale level, these three interaction modes dissipate energy through different relaxation pathways. Hydrogen bonds dissipate energy by reversible rupture and reformation, which favors stretchability and self-recovery but also permits creep. Covalent crosslinks create permanent elastic junctions that improve load transfer and dimensional stability, but they reduce chain mobility when the network is overconstrained. Metal-ion coordination occupies an intermediate regime: ligand exchange and reversible bond rearrangement can dissipate energy and support self-healing, while overly strong ion pairing increases local viscosity and decreases hopping frequency. These distinctions explain why the optimal design is usually a hybrid network rather than a single interaction type.

### 2.3. Effect of Processing History on the Structural Formation of Ionogels

Processing history plays a major role in determining the final network structure of cellulose ionogels. Because cellulose is highly sensitive to the local solvent environment, regeneration pathway, and externally applied orientation conditions, even subtle changes in processing parameters can cause pronounced differences in crystallinity, pore structure, macroscopic anisotropy, and overall network uniformity. These microstructural differences, in turn, strongly affect macroscopic mechanical performance and ion transport. Processing should therefore be treated not as a passive preparation step but as an integral part of structural design.

#### 2.3.1. Construction of Cellulose Skeletons via Dissolution-Regeneration

**Figure 4:**
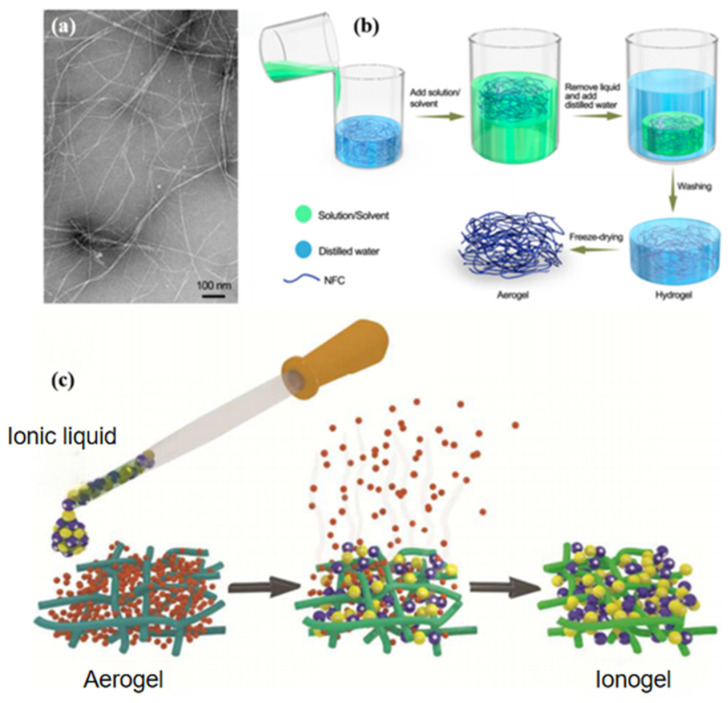
Representative scaffold-construction and stabilization strategies relevant to cellulose ionogels. (**a**) TEM (transmission electron microscopy) image of NFC (nanofibrillated cellulose) showing the building blocks of the hydro/aerogels [51]. (**b**) Schematic representation of the synthesis of NFC hydro/aerogels through solution/solvent exchange treatment. [51]. (**c**) Solvent-exchange fabrication of a bacterial-cellulose ionogel, illustrating the preservation of the preformed fibrillar scaffold during ionic-liquid incorporation [53]. in the schematic, the yellow and purple spheres represent the cations and anions of the ionic liquid, respectively, and the small red spheres represent the solvent molecules removed during solvent exchange.

Dissolution and regeneration are core routes for constructing cellulose ionogels. During dissolution, the native crystalline hierarchy and hydrogen-bonding network of cellulose are disrupted, allowing chains to disperse at the molecular level. Regeneration then drives these chains to re-associate and form the primary load-bearing framework. Solvent exchange is especially important because it controls how the reconstructed network evolves. By changing solvent quality, diffusion kinetics, and polymer–solvent interactions, solvent exchange regulates pore formation, volume shrinkage, and retention of liquid-rich domains. It therefore determines whether the regenerated matrix remains open and continuous or collapses into a dense, heterogeneous structure that restricts ion migration. Previous work has shown that solvent environment, cosolvent concentration, trace impurities, and regeneration conditions all influence the final organization of cellulose materials [23]. At the molecular level, solvent exchange can reorganize hydrogen bonding and chain packing, thereby affecting molecular orientation and mechanical behavior. At the macroscopic level, mild dissolution followed by solvent-mediated reconstruction can induce self-supporting gels in nanofibrillated cellulose systems and produce densely packed porous frameworks with high compressive strength (Figure 4a,b) [51,52]. Similarly, introducing ionic liquids into a preformed bacterial-cellulose network through mild exchange can produce a self-supporting gel with an ionic-liquid content above 99 wt% while preserving the original fibrillar scaffold (Figure 4c) [53].

Taken together, these studies suggest that the formation of regenerated cellulose structures depends not only on chain reassociation but also on how solvent exchange governs subsequent structural fixation and liquid redistribution. Precise control over dissolution, regeneration, and solvent exchange therefore shapes pore evolution, liquid retention, and network continuity, thereby helping balance structural integrity with ionic accessibility in cellulose ionogels.

#### 2.3.2. Construction of Anisotropic Cellulose Skeletons via Directional Assembly

After dissolution and regeneration, cellulose ionogels usually form continuous but randomly arranged isotropic networks. Although such homogeneous structures provide basic stability, they also create a trade-off between mechanical robustness and ion transport. Directional assembly addresses this issue by reorganizing an initially isotropic matrix into an ordered anisotropic structure. Methods such as wet stretching [54,55], shear-induced orientation [56,57] and directional freezing [58,59,60] can align cellulose fibrils, internal pores, and liquid-rich domains. This organization spatially separates mechanical support from ion transport, allowing these functions to operate along different directions rather than within the same homogeneous microphase.

For example, bacterial nanocellulose ionogel films prepared by wet stretching followed by ionic-liquid swelling showed excellent stiffness and mechanical damping while maintaining relatively high ionic conductivity [61]. Directional freezing has also been used to create one-dimensional aligned microchannels within a cellulose hydrogel electrolyte, improving both mechanical robustness and ion-transport efficiency [62]. In template-induced assembly systems, orientation of cellulose-nanofibril-dominated networks markedly improves gel stretchability, ultimate tensile strength, and cyclic fatigue resistance [63].

The main advantage of directional assembly lies in its ability to impart well-defined functional anisotropy. The dense, cellulose-rich framework can withstand substantial mechanical loads along the preferred orientation, while retaining low-resistance ion-transport pathways to support rapid and efficient ionic conduction.

#### 2.3.3. Regulation of Cellulose Skeletons via Post-Treatment

**Figure 5:**
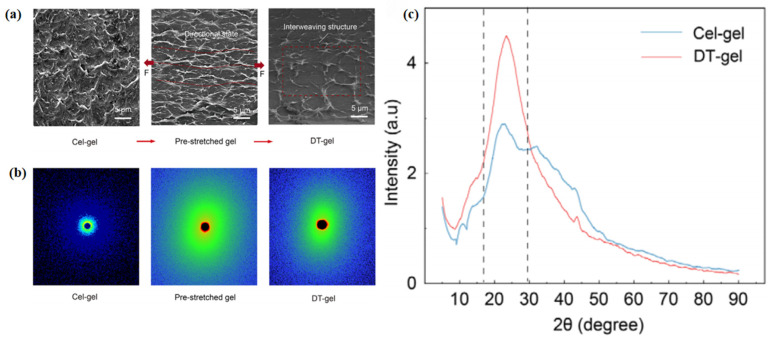
Representative structural evidence for post-treatment-induced densification and reorganization in cellulose ionogels. (**a**) SEM (scanning electron microscopy) comparison of Cel-gel, Pre-stretched gel, and DT-gel, illustrating the transition from a rough and disordered morphology to a more aligned and interwoven structure after secondary treatment. (**b**) Two-dimensional SAXS (small-angle X-ray scattering) patterns demonstrating the increased structural regularity of the post-treated ionogel. (**c**) XRD (X-ray diffraction) profiles of Cel-gel (blue line) and DT-gel (red line), confirming enhanced ordering and a denser network structure after dual treatment [23].

Once a continuous cellulose ionogel network has formed, post-treatment becomes important, especially when the initial matrix contains substantial free volume or loosely packed regions. At this stage, reinforcement depends less on rebuilding the primary network and more on consolidating the existing scaffold. Controlled drying, pressing, or thermal annealing can bring cellulose chains and fibrillar units into closer contact, strengthen intermolecular interactions, and reduce pores that contribute little to mechanical performance. This produces a denser structure with more efficient stress transfer. In regenerated cellulose systems, such densification is widely used to improve compactness and mechanical robustness (Figure 5) [23,64].

A representative example is a nanofibrillated cellulose gel prepared through mild solution treatment and solvent exchange. NaOH-treated samples showed a denser pore structure and significantly higher compressive strength than control samples treated with other solvents, which produced looser structures [54]. This result shows that post-gelation consolidation can improve the mechanical performance of cellulose networks by promoting tighter packing and reducing redundant pore volume. Densification can therefore be regarded as a secondary optimization step that enhances strength and load transfer while preserving the continuous scaffold formed during gelation.

## 3. Synergistic Decoupling of Mechanical Stability and Ionic Conductivity

In cellulose ionogels, mechanical robustness and ionic conductivity are inherently coupled because both properties are governed by the same multiscale network structure. From the perspective of polymer network theory, increasing effective crosslink density, chain orientation, or crystalline reinforcement generally improves modulus, strength, and load transfer. However, these changes may simultaneously reduce mesh size, free volume, and segmental mobility, thereby limiting ion migration. Ionic conductivity can be described by the combined effects of mobile ion concentration, charge, and mobility, where ion mobility is strongly influenced by polymer-chain relaxation, ion association, and the continuity of liquid-rich conductive pathways. Therefore, true performance decoupling requires more than simply strengthening the network; it requires the spatial, dynamic, or directional redistribution of mechanical-supporting domains and ion-transporting domains.

**Figure 6:**
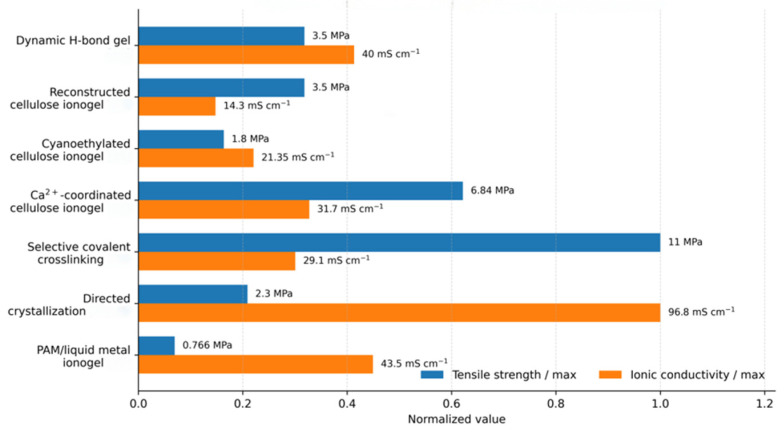
Comparative summary of representative ionogels in terms of tensile strength and ionic conductivity [23,26,39,40,41,65,66,67].

Accordingly, the strategies discussed in this section are classified by their dominant structural level: molecular-level precursor engineering, nanoscale phase-separated networks, mesoscale network design, crystallization-induced reorganization, and macroscopic anisotropic assembly. The following subsections explain how each strategy moves cellulose ionogels away from the conventional trade-off between mechanical reinforcement and ion transport. Representative mechanical and conductive performances are compared in Figure 6.

### 3.1. Precursor Engineering for Performance Decoupling

**Figure 7:**
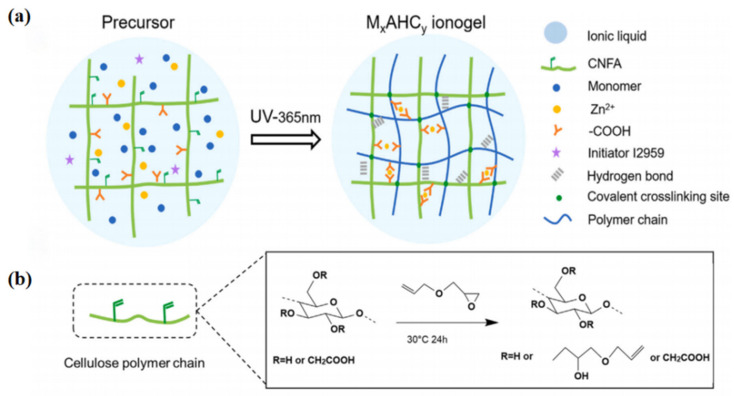
Schematic illustration of precursor engineering for performance decoupling in cellulose-based ionogels. (**a**) Precursor-programmed formation of a multifunctional ionogel, in which modified nanocellulose, ionic liquid, monomers, and dynamic metal ions are integrated prior to polymerization to generate covalent cross-linking sites, hydrogen-bond interactions, and phase-separated soft/hard domains. (**b**) Chemical modification of nanocellulose precursor by allyl glycidyl ether, introducing reactive double-bond moieties that enable macromolecular covalent cross-linking during subsequent network formation [38].

Precursor engineering is an effective way to decouple mechanical robustness from ionic conductivity in cellulose ionogels. The core idea is to tailor the cellulose component before network formation. Instead of uniformly reinforcing the whole matrix, this strategy introduces selected functional groups or reactive motifs so that different structural regions can play different roles. One region may form a load-bearing framework through specific interactions or rigid junctions, while nearby ionic domains remain less constrained and support rapid ion transport. Precursor engineering, therefore, shifts the design from homogeneous reinforcement to selective molecular-scale control of structural units and ionic environments (Figure 7) [38,65,68].

For example, a nanostructured ionogel was prepared through a thiol-ene click reaction between allyl cellulose and POSS-8SH (octamercaptopropyl polyhedral oligomeric silsesquioxane) [69]. The modified precursor introduced rigid POSS-based crosslinking nodes that improved structural stability and promoted microphase separation, creating relatively independent ion-transport pathways. This example shows that precursor modification does more than strengthen the network; it also controls network formation, stress dissipation, and ion migration. Precursor engineering therefore offers a useful route for mitigating the trade-off between mechanical strength and ion-transport capability in cellulose ionogels.

### 3.2. Network Design for Performance Decoupling

At the network level, effective decoupling of mechanical and electrical properties depends not on homogeneous reinforcement but on the rational design of structural heterogeneity. When load-bearing and ionic-conduction functions are confined to the same isotropic network, reinforcing the framework inevitably suppresses ion transport. If these two functions can instead be assigned to interconnected but spatially separated microdomains, the material’s mechanical robustness can be improved while continuous ion-transport pathways are preserved.

#### 3.2.1. Performance Decoupling Based on Phase-Separated Networks

**Figure 8:**
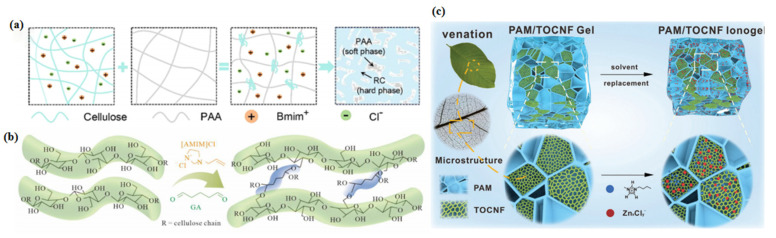
Network design strategies for the mechanical-conductive performance decoupling of cellulose ionogels. (**a**) Schematic illustration of the formation process of the rigid–flexible dual-phase structure with phase separation characteristics [71]. (**b**) Schematic diagram of the catalyst-free chemically cross-linked cellulose network with localized selective cross-linking sites [72]. (**c**) Schematic diagram of the leaf-inspired hierarchical meshing interpenetrating double-network structure, and the toughness comparison of the as-prepared double-network ionogel with other state-of-the-art gel systems [73].

The most direct way to decouple mechanical performance from ion transport is to assign these two functions to different phases. In phase-separated cellulose ionogels, the mechanically robust phase provides structural support, whereas the liquid-rich phase maintains continuous ion-transport pathways. The key advantage of this strategy lies not simply in forming a heterogeneous structure but in preserving continuity in both phases. When phase size, interfacial compatibility, and spatial connectivity are properly controlled, phase separation can reinforce the matrix without interrupting ion migration [17,70]. This principle has been validated in a variety of cellulose-based systems. For example, a sunlight-responsive cellulose ionogel prepared through photoinduced phase separation showed excellent mechanical robustness, strong interfacial adhesion, and good ionic conductivity (Figure 8a) [71,72,73]. These results suggest that, as long as the conductive phase remains continuous, reinforcing the matrix does not necessarily disrupt ion-percolation pathways. Phase separation creates bicontinuous structures where the mechanical phase (e.g., cellulose-rich domains) and conductive phase (ionic-liquid-rich channels) are co-continuous. Theoretical models predict that a double-percolation pathway can maintain high ionic conductivity as long as the conductive phase volume fraction exceeds the percolation threshold (~15–30 vol%), even if the matrix is significantly reinforced. This explains why phase-separated ionogels often outperform homogeneous ones.

#### 3.2.2. Dynamic Selective Cross-Linked Networks for Performance Decoupling

Mechanical performance and ion transport can also be decoupled by localizing net-work constraints. Dynamic and selectively crosslinked networks do not immobilize the entire framework (Figure 8b). Instead, rigid or permanent crosslinking points are placed mainly where structural stability is needed, while adjacent regions retain enough seg-mental mobility for ion transport and local stress relaxation. This creates targeted rein-forcement: some domains bear load and maintain network integrity, whereas others dis-sipate energy and keep conductive pathways open. In cellulose-based conductive net-works, this spatially differentiated constraint is more effective than simply increasing overall crosslinking density [72].

Prepared through a catalyst-free, glutaraldehyde-mediated fixation process, the chemically crosslinked cellulose ionogel features localized covalent crosslinks, enabling a tensile strength of approximately 11 MPa while maintaining an ionic conductivity of 29.1 mS·cm^−1^ [72,74]. These findings suggest that when structural fixation is confined mainly to specific regions, reinforcing the framework does not necessarily compromise ion transport. Further studies have extended this approach by combining nanocellulose-based covalent crosslinking with Zn^2+^-coordinated dynamic junctions in heterogeneous ionogel structures [50]. In these systems, the covalent network provides structural stability, whereas reversible coordination bonds contribute energy dissipation and self-healing. The key design factor is therefore the spatial distribution of crosslinking modes rather than crosslinking density alone. Selective fixation combined with dynamic relaxation enables both mechanical robustness and ionic accessibility.

#### 3.2.3. Double-Network Architectures for Performance Decoupling

Interpenetrating networks and complex topologies extend performance decoupling from local molecular interactions to the mesoscale. By assigning distinct functions to different network components, these designs overcome the limitations of homogeneous reinforcement within a single continuous matrix. In cellulose ionogels, the primary network usually acts as a rigid load-bearing scaffold or sacrificial network, whereas a secondary flexible phase is introduced to maintain efficient ion transport. Mechanical deformation, energy dissipation, and ionic percolation are therefore distributed across multiple cooperative structural elements rather than being concentrated within a single structural unit. As a result, the macroscopic properties arise more from topological synergy than from a simple increase in crosslinking density. Existing studies on cellulose-based electrolytes have widely recognized double-network, interpenetrating-network, and topologically entangled architectures as representative strategies for combining mechanical toughness with rapid ion transport [65,75].

In one representative study, polyacrylamide was introduced into TEMPO-oxidized (2,2,6,6-tetramethylpiperidine-1-oxyl) cellulose nanofibril clusters and combined with solvent exchange to produce a mechanically robust gel with a high ionic-liquid content (Figure 8c) [73]. In this system, the network topology promoted cooperation between rigid cellulose-rich clusters and the flexible synthetic polymer matrix, allowing the material to reach a fracture strength of 6.5 MPa. A similar decoupling mechanism has also been demonstrated in a semi-interpenetrating-network ionogel constructed from phosphorylated straw cellulose and a polyaspartamide derivative [76]. In that system, the cellulose subnetwork primarily provided structural reinforcement, whereas the second polymer network preserved deformability and ionic conductivity.

Overall, the key advantage of interpenetrating-network and topological designs lies in their ability to maintain continuous connectivity in both mechanically dominant and transport-dominant domains without requiring structural homogenization.

### 3.3. Crystallization-Induced Structural Reorganization for Performance Decoupling

Crystallization can decouple performance by selectively reorganizing the cellu-lose-rich framework without imposing a uniform constraint on ion transport. Crystalliza-tion-induced reconstruction offers two main benefits. First, it promotes ordering and den-sification in the load-bearing phase by removing excess free volume and strengthening in-terchain interactions. Second, it avoids rigidly constraining the whole material, allowing ionic conduction to remain efficient in continuous liquid-rich pathways. This strategy therefore provides a useful microstructural model for regenerated cellulose-based multi-functional materials. For example, a cellulose ionogel was constructed using a LiBr/ZnCl_2_/H_2_O solvent system. During cooling, local ionic crystallization brought dis-solved cellulose chains closer together and generated a dense, oriented framework. Sub-sequent controlled moisture uptake transformed this crystalline precursor into a flexible gel matrix. Part of the crystalline constraint was released, while hydrated ions and cellu-lose chains rebuilt the network through ionic complexation and hydrogen bonding. The resulting material exhibited a tensile strength of 2.3 MPa and an ionic conductivity of 96.8 mS·cm^−1^, showing that directed crystallization can consolidate the load-bearing cellulose framework without disrupting the internal ion-transport phase [26,66].

### 3.4. Anisotropic Reorganization for Performance Decoupling

Anisotropic organization is an effective decoupling strategy because it prevents the cellulose framework from having to serve simultaneously as both a mechanical support and an ion-transport medium along the same spatial direction. Through uniaxial or multiaxial orientation, such structures redistribute these functions: the aligned cellulose-rich framework primarily bears and dissipates mechanical loads along selected directions, whereas continuous liquid-rich microchannels provide parallel pathways for rapid ionic transport. In cellulose ionogels, the importance of inducing anisotropy therefore lies not simply in achieving greater macromolecular order, but in spatially separating the reinforcement mechanism from the transport mechanism. Studies on cellulose-based electrolytes have consistently shown that precise orientation and hierarchical organization can impart directional ion-transport behavior while preserving good mechanical robustness.

Several representative studies demonstrate this design principle. Bacterial nanocellulose ionogel films prepared by combining wet stretching with ionic-liquid swelling exhibited excellent stiffness and mechanical damping without a substantial loss of ionic conductivity [77]. Building on this concept, an oriented cellulose nanofibril ionogel with continuous micro- and nanochannels was developed through directional freezing, thereby creating low-tortuosity transport pathways that facilitate rapid, directional ion migration [78]. Taken together, these studies indicate that anisotropic organization of the cellulose-rich phase can optimize mechanical support and ion transport along different, often orthogonal, directions, thereby helping overcome the intrinsic trade-off found in isotropic polymer networks. This comparison indicates that cellulose ionogels should not be evaluated only by peak conductivity or maximum stretchability (Table 2). Synthetic organic ionogels commonly offer lower hysteresis and larger reversible deformation because their soft polymer backbones are highly mobile, whereas cellulose ionogels more often provide higher modulus, better thermal stability, and renewable structural scaffolds. Therefore, cellulose-based systems are particularly competitive in devices requiring dimensional stability, environmental tolerance, and sustainable materials, while they still need improvement in high-frequency response, electrode integration, and scalable patterning.

The advantages, limitations, and suitable design conditions of these decoupling strategies are summarized in Table 2.

## 4. Emerging Applications of Cellulose Ionogels in Flexible Electronics

As cellulose ionogel research matures, the design paradigm is shifting from optimizing isolated material properties to application-specific structural design. For next-generation flexible electronics, the main goal is no longer to maximize mechanical strength or ionic conductivity in isolation. Instead, materials must provide stable signal transmission, robust electrochemical performance, and sustained structural integrity under practical operating conditions. Although cellulose ionogels are promising, comparison with state-of-the-art organic ionogels is necessary to identify current gaps, as summarized in Table 3.

### 4.1. Flexible Sensors

For flexible sensors, cellulose ionogels offer a particularly attractive balance between mechanical stability, ionic signal transduction, and environmental reliability. A wearable sensor layer must remain conformal to skin, preserve continuous conductive pathways during repeated bending or stretching, and avoid signal drift caused by dehydration, freezing, leakage, or irreversible network damage. Cellulose ionogels address these requirements through a dual-phase structural logic: the cellulose-rich framework acts as a renewable and mechanically stable scaffold, while the ionic-liquid-rich phase provides nonvolatile ionic conduction. Compared with water-swollen hydrogels, this design reduces the dependence of sensing performance on water retention; compared with many purely synthetic ionogels, the hierarchical cellulose network offers additional load transfer, interfacial anchoring, and sustainability advantages [81,82,83,84,85,86].

**Figure 9:**
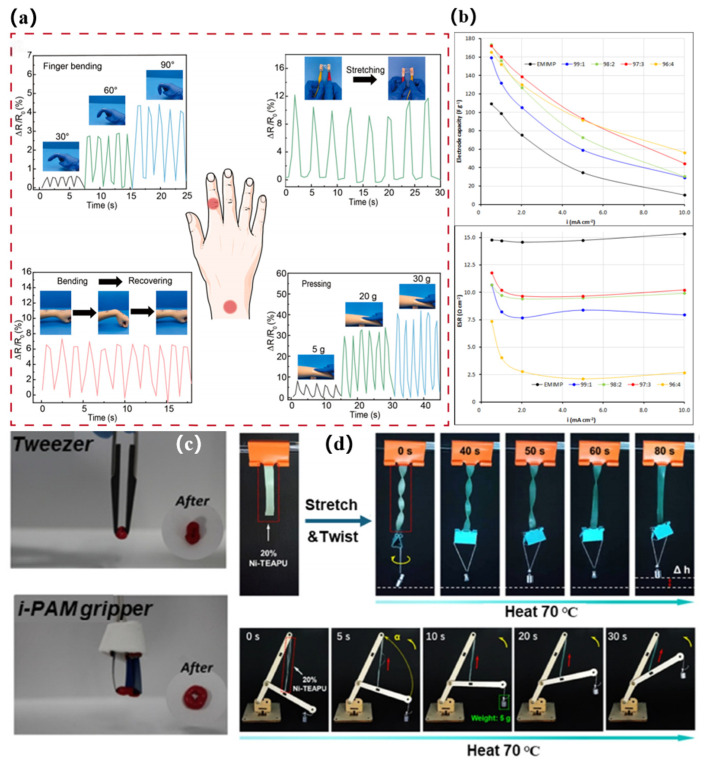
Representative applications of cellulose-based ionogel systems in flexible electronics and soft actuators. (**a**) Multimodal sensing performance, including real-time resistance responses to finger bending at different angles, stretching, bending-recovery cycles, and pressure loading with different weights, demonstrating the sensitivity and operational stability of the ionogel sensor [74]. (**b**) Electrode capacity and equivalent series resistance (ESR) of supercapacitors employing EMIMP (1-ethyl-3-methylimidazolium dimethyl phosphate) and EMIMP/CNFc (EMIMP/carboxymethylated cellulose nanofibers) ionogel electrolytes at different current densities [92]. (**c**) Representative gripping/manipulation behavior of a thermoresponsive ionogel actuator in tweezer- and gripper-type configurations [93]. (**d**) Heat-triggered shape recovery, contraction, and controllable load-lifting performance of thermoresponsive ionogel-based soft actuators [93]. The arrows in panel (**d**) indicate the direction of the applied force during mechanical deformation.

Recently developed cellulose-ionogel sensors demonstrate this balanced advantage. A cellulose-enhanced double-network ionogel sensor showed a tensile strength of 0.48 MPa, an elongation at break of about 900%, adhesion of 47.18 kPa, autonomous self-healing, and reliable responses to human motion and temperature changes [87]. More importantly, strong cellulose reinforcement does not necessarily block ion transport. For example, a catalyst-free chemically crosslinked cellulose ionogel reached a tensile strength of approximately 11 MPa, a toughness of 2.8 MJ·m^−3^, and an ionic conductivity of 29.1 mS·cm^−1^, enabling pressure sensing, body-motion monitoring, health monitoring, and Morse-code communication [72]. A self-crosslinked cellulose ionogel further combined high tensile strength (4.23 MPa), toughness (1.36 MJ·m^−3^), optical transmittance (93%), and desirable biodegradation (Figure 9a) [74]. A corncob-cellulose-derived ionogel sensor also retained 1.28 MPa tensile strength, 573% elongation, thermal stability up to 278 °C, flexibility at −25 °C, and a gauge factor of 1.23–2.08 for subtle-strain and ECG monitoring [88]. These results show that cellulose is not merely a passive biofiller in sensing gels; it can act as the load-bearing skeleton, interfacial stabilizer, and structural regulator of ionic pathways.

PVA- and PAM-based ionic gels are useful reference materials for evaluating cellulose ionogels in flexible sensors because they are widely studied, easy to process, and can provide good softness, transparency, stretchability, and sensing sensitivity. For example, a PVA/tannic acid/LiCl hydrogel sensor showed 0.1 MPa tensile strength, 0.64 S·m^−1^ conductivity, a gauge factor of 1.15, low hysteresis, and stable output over 2500 tensile cycles [89], indicating its suitability for soft skin-contact sensing; PAM/xanthan gum/sodium alginate/LiCl hydrogels reached fracture strains up to 1800% and a gauge factor of 4.56 [90], while starch/PAM ionogel-type systems containing imidazolium ionic liquids achieved 515.4% elongation, 3.1 S·m^−1^ conductivity, and a gauge factor of 9.3 [91]. These results show that PVA- and PAM-based systems are attractive when softness, high stretchability, or high sensitivity is the primary target. However, their sensing performance generally relies on hydrated salt-transport channels, water-retention design, plasticizers, or multiple polymer components, which may make dimensional stability, baseline drift, and environmental durability more difficult to control under dry, cold, or thermally variable conditions. In contrast, cellulose ionogels are not necessarily designed to maximize stretchability or gauge factor alone; their stronger advantage lies in integrating MPa-level mechanical robustness, stable nonvolatile ionic conductivity, optical transparency, adhesion or interfacial compatibility, environmental tolerance, and renewable material origin within one platform. Therefore, compared with PVA- and PAM-based sensing gels, cellulose ionogels are particularly promising for wearable ionotronic sensors that require repeated mechanical loading, long-term signal stability, capacitive or resistive readout, and reliable operation under variable humidity and temperature. Future work should further reduce hysteresis, improve low-strain resolution, and strengthen electrode–gel integration so that the intrinsic structural advantages of cellulose ionogels can be fully translated into device-level reliability [92,93].

### 4.2. Solid-State Electrolytes for Flexible Supercapacitors

In flexible supercapacitors, the electrolyte serves both as an ion-conducting medium and as a mechanical separator. In addition to supporting efficient ion transport, it must prevent short circuits between electrodes, suppress liquid leakage, and maintain intimate interfacial contact during repeated deformation such as bending, twisting, and folding. High ionic conductivity alone is therefore insufficient for practical use. Mechanical integrity, dimensional stability, and interfacial robustness are equally important because they determine whether the material can sustain rapid ion-transport kinetics without electrolyte failure, interfacial delamination, or electrical shorting. For this reason, studies on gel polymer electrolytes and ionogels have consistently emphasized that combining liquid-like ion mobility with solid-like mechanical toughness is essential for the stable operation of flexible energy-storage devices [86,94,95].

Cellulose ionogels meet these requirements because they integrate ion conduction and mechanical separation in one material platform. The cellulose-rich framework confines the ionic-liquid phase, reducing leakage, improving structural reliability, and preserving separator integrity during deformation. The ionic-liquid phase provides continuous ion-transport pathways, a wide electrochemical window, and negligible volatility. This combination is attractive for flexible supercapacitors, where electrochemical performance depends on both electrolyte conductivity and mechanical stability under stress [84]. Compared with petroleum-derived gel electrolytes, cellulose ionogels offer renewability, leakage resistance, thermal stability, and structural robustness. However, their processing reproducibility, pore-structure control, and electrode compatibility remain less mature than those of many synthetic polymer systems.

Beyond typical ionic-liquid-based cellulose ionogels, acid- or alkali-doped cellulose and nanocellulose membranes provide another related route for constructing flexible ion-conducting separators for supercapacitors. In such systems, diluted acids or concentrated alkali solutions, such as 6 M NaOH or KOH, can induce swelling, partial ionization of cellulose hydroxyl groups, and mercerization-related structural transformation from Cellulose I to alkali-cellulose intermediates and then to Cellulose II. For example, KOH-doped bacterial nanocellulose and nanocellulose-impregnated polybenzimidazole membranes have been reported as robust flexible separators for supercapacitor cells [96]. Compared with ionic-liquid-based cellulose ionogels, these alkali-doped cellulose membranes are attractive because they preserve a relatively stable nanofibrillar framework and can provide good separator integrity. However, they are not typical ionogel systems in which ionic liquids are confined within a soft polymer network, and their ion-transport behavior, environmental stability, and mechanical response under repeated bending or stretching are governed by different mechanisms. Therefore, they can be regarded as useful reference systems for flexible energy-storage applications, while cellulose ionogels remain particularly promising when nonvolatility, leakage resistance, wide electrochemical stability, and mechanical–conductive property decoupling are required simultaneously.

The viability of this dual advantage is exemplified by a double-network ionogel electrolyte based on phosphorylated cellulose. This material exhibits a highly tunable ionic conductivity ranging from 2.6 to 22.4 mS·cm^−1^ coupled with a mechanical toughness of up to 1.46 MJ·m^−3^. When integrated as a separator electrolyte within a flexible supercapacitor, the assembled device achieves a specific capacitance of 174 F·g^−1^. Notably, it retains 88% of its rate capability even under stringent operating conditions of 120 °C and 2.5 V [97]. Incorporating carboxymethylated cellulose nanofibers into an ionic liquid gel matrix simultaneously enhances printability and reduces the equivalent series resistance. This structural modification effectively increases the device capacitance from 99 F·g^−1^ to 160 F·g^−1^ (Figure 9b) [92]. These results indicate that rational structural design of the cellulose phase can directly improve electrochemical utilization efficiency by simultaneously stabilizing the electrolyte framework and facilitating ion migration.

Ultimately, the superiority of cellulose ionogels in flexible supercapacitors stems from the synergistic integration of mechanical robustness and ionic conductivity, rather than the optimization of either property in isolation. Because the cellulose framework stabilizes the ionic-liquid phase without hindering ion migration, these ionogels offer the mechanical reliability, interfacial durability, and rapid ion transport demanded by high-performance flexible energy-storage devices.

### 4.3. Soft Actuators and Robotics

Soft actuators place particularly demanding requirements on ion-conducting gels because ion transport in these systems not only provides electrical conduction but also directly drives electromechanical response. Ion-transport capability and electrochemical stability determine response speed, operating voltage, and actuation reversibility, whereas mechanical strength, elastic modulus, and fatigue resistance determine whether localized ionic redistribution can be translated effectively into macroscopic deformation, blocking force, and long-term operational stability without creep or structural failure. Studies on soft ionotronic actuators have therefore widely identified ultralow-voltage actuation, cyclic durability, and efficient electromechanical conversion as key performance requirements for soft-robotic applications (Figure 9c,d) [93,98,99].

Cellulose ionogels are advantageous for soft actuators because they integrate ionic conductivity and mechanical support within one platform. The cellulose-rich framework reinforces the actuator body and preserves structural integrity during repeated deformation, while the confined ionic-liquid phase provides a nonvolatile, continuous pathway for ion transport and enables low-voltage, stimulus-responsive actuation. This structure is especially useful because actuator performance depends on both rapid ion migration and the ability of the matrix to maintain dimensional stability during repeated loading. Compared with petroleum-based elastomeric ionogels, cellulose ionogels are more sustainable and structurally stable, although they may show lower stretchability, slower response, and less programmable elasticity. Their main value therefore lies in robust, low-voltage, and environmentally stable soft robotic systems.

A natural cellulose-based ionogel actuator was able to achieve reversible bending under step voltages of only ±500 mV, while exhibiting rapid actuation kinetics and stable electrochemical behavior below 2.5 V [100]. In another study, an ionic soft actuator constructed from cellulose nanofibers, cellulose nanocrystals, and an ionic liquid achieved a maximum tip displacement of 10.2 mm at 1.0 V and retained 95.2% of its operational reliability after 2000 actuation cycles [101]. These results indicate that the value of cellulose ionogels in soft actuators lies not in independently improving conductivity or merely strengthening the matrix, but in coupling rapid ionic responsiveness with sufficient mechanical durability to enable repeatable, efficient actuation.

Overall, the advantage of cellulose ionogels in soft robotic systems lies in the synergistic optimization of ion dynamics and mechanical toughness. When the cellulose framework can support deformation without excessively restricting ion migration, these materials are likely to provide the low-voltage responsiveness, cyclic stability, and electromechanical reliability required for next-generation soft actuators.

## 5. Conclusions and Outlook

Cellulose ionogels have emerged as promising soft functional materials for flexible electronics because they combine the renewable, hierarchically tunable structure of cellulose with the nonvolatile ion-transport capability of ionic liquids. The key conclusion of this review is that the mechanical-conductive trade-off should be treated as a multiscale structural problem rather than a simple materials-selection problem. True decoupling requires mechanical reinforcement to be introduced without proportionally restricting ion mobility. This can be achieved when load-bearing cellulose-rich domains, sacrificial energy-dissipation units, and liquid-rich ion-transport pathways are spatially or dynamically coordinated.

The comparative analysis suggests that different design strategies are suitable for different performance targets. Precursor engineering is most useful when molecular-level functionality and interfacial control are needed. Phase-separated and double-network systems are more effective when simultaneous toughness and ion percolation are required. Selective crosslinking is advantageous for high-strength ionotronic devices, whereas crystallization-induced and anisotropic reorganization are particularly useful for creating ordered load-bearing skeletons or directional ion highways. However, these strategies also have limitations, including possible hysteresis, processing complexity, interfacial instability, and difficulty in maintaining reproducible morphology during scale-up.

Several challenges should be prioritized in future work. First, standardized testing protocols are needed so that tensile strength, toughness, conductivity, hysteresis, fatigue life, and electrochemical stability are reported under comparable temperature, humidity, strain, and frequency conditions. Second, multiscale characterization and modeling should be integrated to quantify how crystallinity, phase continuity, crosslink density, and segmental dynamics determine ion mobility. Third, application-driven design must address electrode adhesion, ionic-liquid leakage, long-term cycling, biocompatibility, recyclability, and manufacturability. Fourth, real-device integration should move beyond material demonstrations toward patterned, miniaturized, and scalable electronic systems. Addressing these challenges will help cellulose ionogels evolve from promising laboratory materials into practical sustainable platforms for wearable sensors, flexible energy storage, and soft robotic devices.

## Figures and Tables

**Table 1:** Comparative Analysis of Three Network Structures in Cellulose Ionogels.

Network Type	Core Mechanism	Mechanical Advantages	Limitations	References
Hydrogen Bond Network	Reversible physical cross-linking via hydroxyl groups; dynamic break-recombination for energy dissipation	High stretchability, self-recovery, and favorable low-temperature tolerance	Creep, poor dimensional stability at high ionic liquid content	[39,40,41,42]
Covalently Cross-Linked Network	Permanent covalent bonding to anchor a 3D framework, formed by irradiation or dynamic covalent chemistry	High structural stability, anti-creep, excellent dimensional retention	Excessively high cross-linking density causes brittleness and restricts segmental motion	[43,44]
Metal-Ion-Coordinated Network	Reversible coordination between multivalent ions and cellulose hydroxyl groups; intermediate strength between physical and covalent cross-linking	Tunable mechanical strength, good stability and dynamics, excellent flexibility at extreme temperatures	Excessively strong coordination constrains the network and hinders ion migration	[49,50]

**Table 2:** Critical comparison of decoupling strategies for cellulose ionogels.

Strategy/Structural Level	Representative Performance	Major Advantages	Suitable Design Condition	Reference
Precursor engineering/molecular scale	3.29 MPa strength; 13.15 MJ·m^−3^ toughness; 975% strain	Programs reactive sites and improves interfacial stress transfer	Molecular functionalization for toughness, stretchability, and interface control	[38]
Heterogeneous network design/phase separation and selective crosslinking	About 11 MPa strength; 29.1 mS·cm^−1^ conductivity	Separates load-bearing domains from conductive liquid-rich pathways	Molecular functionalization for toughness, stretchability, and interface control	[71,72]
Crystallization-induced reorganization/ordered cellulose skeleton	2.3 MPa strength; 96.8 mS·cm^−1^ conductivity	Orders and strengthens the cellulose-rich skeleton	High conductivity with moderate mechanical stability	[26,66]
Anisotropic reorganization/macro-mesoscale orientation	Retained conductivity with higher stiffness, damping, or directional transport	Assigns support and ion transport to preferred directions	Directional strain, bending, or ion-flux devices	[77,78]

**Table 3:** Performance comparison of cellulose-based ionogels with state-of-the-art synthetic organic ionogels in flexible electronics.

Polymer Matrix	Tensile Strength (MPa)	Elongation at Break (%)	Conductivity/Core Sensing Metric	Limitations	Key Advantages	Reference
PAM/Liquid Metal network	0.766	907	43.5 mS/cm	Lower modulus compared to rigid natural polymers	Ultra-stretchable, excellent anti-freezing (−40 °C)	[67]
S-ionogel (Modified Cellulose)	1.8	>990	Seebeck coefficient ~68 mV·K^−1^	Focuses on heat harvesting, specific ionic conductivity not maximized	Rubber-like stretchability, skin-like modulus (63 kPa)	[41]
PVA (poly (vinyl alcohol))/Lignin-based network	~0.036	–	Sustained release and ROS (reactive oxygen species) scavenging	Relatively low tensile strength for extreme mechanical loading	High compressive toughness, excellent bioactivity (antibacterial)	[79]
Collagen/PHEA (PH@Ag)	Robust (Skin-like)	–	30.6 mS/cm	Environmental stability highly depends on moisture retention	Outstanding cytocompatibility, good sensing sensitivity	[80]

Note: Data compiled from referenced studies; “–” indicates not reported.

## Data Availability

No new data were created or analyzed in this study.
